# Accidental Strangulation with Cervical Nerve Root Injury Caused by the Entrapment of Clothing in a Soybean Milling Machine

**DOI:** 10.1155/2019/4706278

**Published:** 2019-02-05

**Authors:** Kazuhiko Omori, Ikuto Takeuchi, Youichi Yanagawa

**Affiliations:** Department of Acute Critical Care Medicine, Shizuoka Hospital, Juntendo University, Japan

## Abstract

The clothing of a forty-five-year-old man became entrapped by the mixing rotator while he was operating a soybean milling machine. His clothing was wound around the rotator, and tightened around his neck and chest, causing strangulation and a loss of consciousness. He was rescued by his coworkers and transported to our hospital by helicopter. Upon arrival, he regained consciousness. A physiological examination revealed multiple petechiae on his face and strangulation marks with subcutaneous hemorrhage on his neck and upper trunk. In addition, he had motor weakness of the right upper extremity and bilateral paresthesia from C5 to Th1. All radiological studies were negative. He was admitted for observation. After the patient's creatine phosphokinase level peaked and his focal neurological signs improved, he was discharged on foot on the 6^th^ hospital day. Accidental ligature strangulation with cervical nerve root injury, in which an article of clothing is caught in an electrical machine and strangles the wearer, is very rare. This case is presented for its rarity and the unique pattern of injury.

## 1. Introduction

Soybean milling machines use a mixing rotator to make sweet soybean paste from soybeans, sugar, and water. Entrapment of an arm or leg by the mixing rotator has been reported to result in degloving injury, or the projection of the blade attached to the rotator has been reported to result in penetrating or cutting wounds [1–3]. Hamajima et al. reported that milling and crushing machines are involved in approximately 10 fatal occupational accidents and approximately 250 accidents involving at least 4 days of lost work time, in Japan each year. However, there were no concrete descriptions how the milling machine had injured workers [4]. We herein report a near fatal case of accidental strangulation with cervical nerve root injury due to the entrapment of clothing in a soybean milling machine.

## 2. Case Presentation

**Figure 1:**
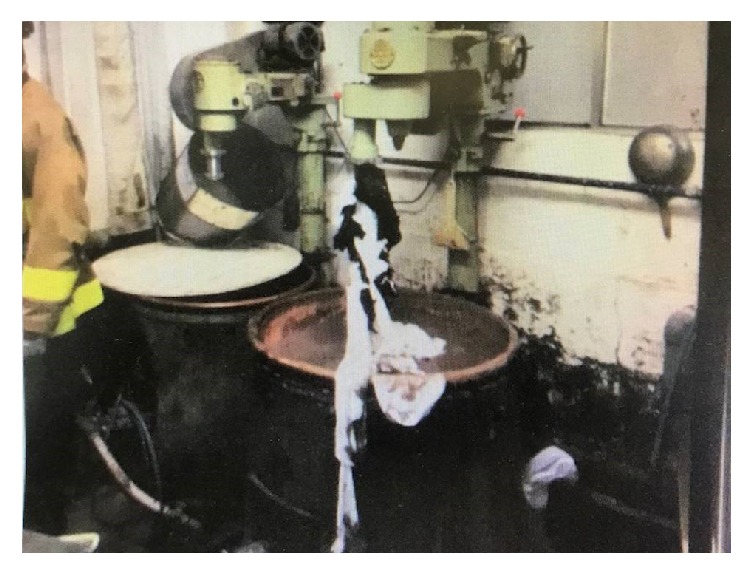
The scene of the accident. The patient's clothing was entrapped and wound around the mixing rotator.

**Figure 2:**
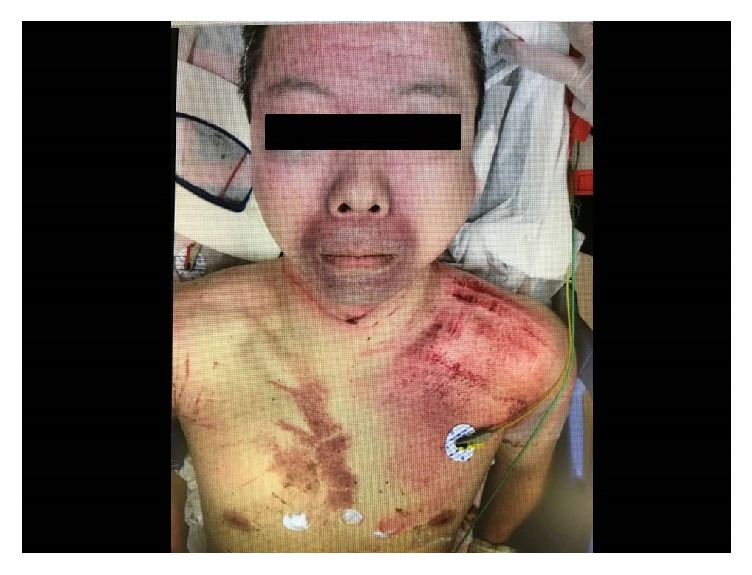
Patient's appearance. A physiological examination revealed multiple petechiae on the patient's face, and strangulation marks with subcutaneous hemorrhage on his neck and upper trunk.

The patient was a forty-five-year-old man who worked at a soybean paste making factory. He had no specific past or family history. While working with a soybean milling machine, his clothing became entrapped by the mixing rotator (Figure 1). His clothing was wound around the rotator and tightened around his neck and chest, causing strangulation and a loss of consciousness. He was rescued by coworkers and transported to our hospital by a physician staffed helicopter. Upon arrival, his vital signs were as follows: Glasgow Coma Scale, E4V4M6; blood pressure, 128/80 mmHg; pulse rate, 78 beats per minute; respiratory rated, 16 breaths per minute, peripheral oxygen saturation on 10 liters of oxygen per minute with a reservoir mask, 100%; and temperature, 35.8°C. A physiological examination revealed multiple petechiae on his face and strangulation marks with subcutaneous hemorrhage on his neck and upper trunk (Figure 2). In addition, he had motor weakness of the right upper extremity and bilateral paresthesia from C5 to Th1. Chest roentgenography, electrocardiography, whole body computed tomography, and cervical magnetic resonance imaging revealed no specific findings. The results of blood biochemical analyses on arrival revealed leukocytosis (16,800/*μ*L) and rhabdomyolysis (creatine phosphokinase, 723 IU/L). He was admitted for observation. After the patient's creatine phosphokinase level peaked and his focal neurological signs improved, he was discharged on foot on the 6^th^ hospital day.

## 3. Discussion

Cases of accidental strangulation caused by clothing are extremely rare; this is the rare case report of a patient with accidental strangulation with cervical nerve root injury caused by the entrapment of clothing in a soybean milling machine.

Strangulation is generally observed in cases of homicide; in cases of accidental strangulation circumstantial evidence alone might point toward the accidental nature of the incident. Arun et al. reported a rare fatal accident in which the deceased individual was strangled by the heated rubber belt of a rice milling machine [5]. To the best of our knowledge, there are two previous reports on accidental strangulation by clothing in the English literature. Dogan et al. reported a case in which a woman's headscarf was caught on a cylinder ironing machine in the laundry of the hospital in which she worked. The autopsy revealed that death occurred as a result of accidental ligature strangulation [6]. Parchake et al. reported that, while working in agricultural field, a woman's saree (a garment traditionally worn by women in India) became entangled in a crop threshing machine, causing strangulation. She was taken to the nearest hospital where she died. The autopsy revealed a cross ribbon-shaped ligature mark on the neck and anterior chest, similar to the present case [7].

Cervical nerve root injury in survivors of accidental strangulation is a rare event. Strangulation can injure the soft tissues of the neck; the larynx, trachea, esophagus, and cervical spine; and the nerves located in the neck, which include the laryngeal, facial, and phrenic nerves [8, 9]. These injuries may not be immediately apparent [10]. We could not find any literature that reported bilateral paresthesia from C5 to Th1 after strangulation. However, the nerves from C5 to Th1, which form the brachial plexus, are located in the neck. Accordingly, nerve injury might be part of the pathophysiological mechanism of strangulation and hanging, and a clinical investigation to exclude nerve injury should be considered for patients who survive such events.

To reduce the number of fatal milling machine-related accidents, the accumulation of concrete data concerning how accidents involving milling machine occur will be necessary in the future. At a minimum, a protective fence should be installed around the rotator of the machine and workers should be asked to wear close-fitting clothing as safety measures.

## 4. Conclusion

This is the rare case report of accidental strangulation with cervical nerve root injury caused by the entrapment of clothing in a soybean milling machine. In the future, it will be necessary to accumulate concrete data on how milling machine-related accidents occur in order to reduce the number of fatal milling machine-related accidents.
